# Skullcap (*Scutellaria baicalensis*) Hexane Fraction Inhibits the Permeation of Ovalbumin and Regulates Th1/2 Immune Responses

**DOI:** 10.3390/nu9111184

**Published:** 2017-10-28

**Authors:** Sun Young Jung, So-Young Lee, Dae Woon Choi, Hye-Jeong See, Da-Ae Kwon, Jeong-Ryong Do, Dong-Hwa Shon, Hee Soon Shin

**Affiliations:** 1 Food Biotechnology Program, Korea University of Science and Technology, Daejeon 34113, Korea; Jung.Sun-young@kfri.re.kr (S.Y.J.); sylee09@kfri.re.kr (S.-Y.L.); choidw19@gmail.com (D.W.C.); 2 Division of Nutrition and Metabolism Research, Korea Food Research Institute, 1201-62, Anyangpangyo-ro, Bundang-gu, Seongnam-si, Kyeonggi-do 463-746, Korea; jjung3015@naver.com (H.-J.S.); ksloveda@naver.com (D.-A.K.); jrdo@kfri.re.kr (J.-R.D.); dhs95@kfri.re.kr (D.-H.S.)

**Keywords:** skullcap, *Scutellaria baicalensis*, linoleic acid, Th1/Th2 balance, Th1/2 immune response, ovalbumin permeation

## Abstract

Skullcap (*Scutellaria baicalensis*) is well known for its anti-inflammatory and anti-allergic effects. In our previous study, we found that skullcap could inhibit allergen permeation and regulate Th1/2 immune balance. To reveal the key fractions and components of skullcap, we fractionated skullcap extract into five fractions: hexane, chloroform, ethyl acetate, butanol, and water fraction. Among these fractions, the hexane fraction significantly suppressed the production of Th2-mediated cytokines (Interleukin (IL)-4, 5, 10 and 13) and increased Th1-mediated cytokines (Interferon (IFN)-γ and IL-12). Furthermore, the hexane fraction inhibited the permeation of ovalbumin (OVA), used as an allergen, across the intestinal epithelial cell monolayer. To confirm the active compounds in the hexane fraction, fatty acids were analyzed. Linoleic acid (LA, C18:2 (>59.7%)) was identified as the most important fatty acid in the skullcap hexane fraction. LA significantly suppressed IL-4 production and increased IFN-γ secretion, as well as inhibiting OVA permeation. Thus, LA significantly diminished the permeation of allergen by enhancing intestinal barrier function and regulated allergic responses to maintain Th1/Th2 immune balance.

## 1. Introduction

Skullcap (*Scutellaria baicalensis*) is widely used across Asia as an alternative herbal medicine owing to its anti-inflammatory, anti-allergic, and anti-viral activities [1,2]. It contains many active components, such as baicalin, baicalein, and wogonin [2,3]. These components may contribute to the physiological functionalities of skullcap. In a previous study, skullcap extract inhibited the permeation of ovalbumin (OVA) by upregulating the expression of tight junction (TJ) proteins, and the active component baicalein enhanced intestinal barrier function [4]. Furthermore, wogonin suppressed Th1 and Th2 responses without influencing cell viability, and its effect was similar to that of skullcap ex vivo and in vivo [5]. However, there are cases in which the intact extract does not exhibit these physiological functionalities, although the active functional components are present in the extract. For example, baicalin and baicalein, the main active components, can induce regulatory T (Treg) cells, resulting in suppression of Th1, Th2, and Th17 immune responses; however, skullcap extract is not able to regulate the differentiation of Treg cells [6,7]. The baicalein or baicalin, as a single compound, has important implications, because it is able to treat autoimmune disorders through the induction of Treg cells. To identify the potential active components in the extract, the fractionation, component analysis, and functional evaluation of the extract are essential. Therefore, in this study, we focused on the fractions of skullcap extract to reveal novel functions and to identify the active components in the fractions.

In general, the immune system maintains Th1 and Th2 immune balance in normal conditions. However, in one study, Th2 immune responses increased and Th1 responses decreased when allergens triggered allergic immune responses [8]. Anti-allergic agents can reverse this immune imbalance. In our previous study, we revealed that skullcap extract was able to modulate Th1/Th2 immune balance in a mouse food allergy model [9]. Thus, we investigated the potential components in the skullcap extract that could regulate Th1 and Th2 immune balance under allergic conditions.

Intestinal epithelium has important functions, such as absorbing dietary nutrients and blocking the permeation of noxious substances (allergens). Allergens permeate the body through the paracellular diffusion pathways in the intestinal epithelium and induce allergic responses [10]. If the allergen permeation can be suppressed, the allergic immune responses can be attenuated. Therefore, we examined whether the components in the skullcap extract inhibited allergen permeation into human intestinal epithelial Caco-2 cell monolayers [10,11,12].

The aim of this study was to identify the key components and fractions of skullcap extract and investigate their effects on Th1/Th2 immune balance and allergen permeation across intestinal epithelium.

## 2. Materials and Methods

### 2.1. Animals

Female BALB/c mice (weight, 16–18 g) were purchased from Orient Bio Inc. (Kyeonggi, Korea). The 5-week-old mice were housed in an air-conditioned room (23 ± 2 °C) with a 12-h light/dark cycle. They were allowed free access to food and tap water. All animal experiments were performed according to the guidelines for animal use and care at the Korea Food Research Institute. (permit number: KFRI-M-16029).

### 2.2. Sample Preparation

**Figure 1:**
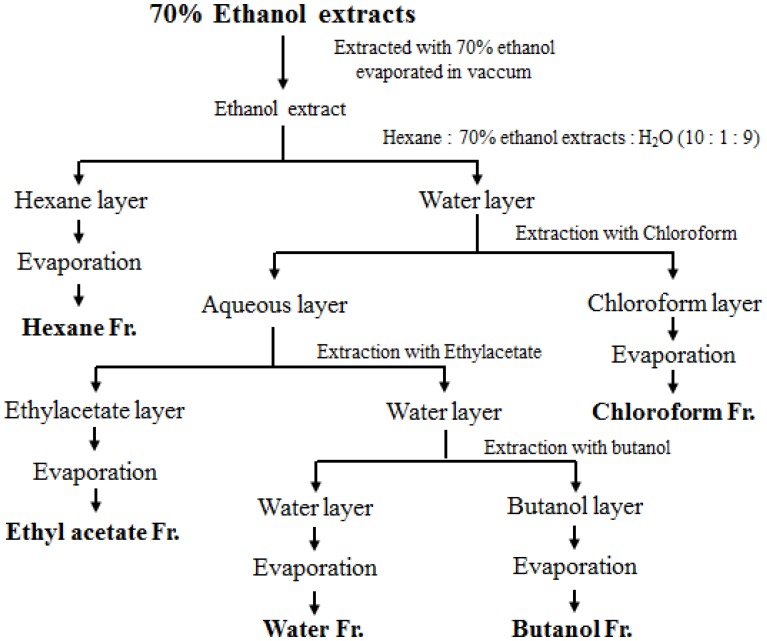
Solvent fractions of skullcap with ethanol, hexane, ethyl acetate, chloroform, butanol, and water.

The skullcap used in this study was purchased from Kyeong-dong Oriental Pharmacy (Seoul, Korea) and identified by Professor Y. Bu, Department of Herbal Pharmacology, Kyung Hee University. The specimen (KFRI-SL-101) was stored at the Functional Materials Research Group, Korea Food Research Institute. The skullcap sample was obtained by reflux extraction in 70% ethanol using a Soxwave 100 apparatus (Prolabo, Paris, France). The ethanol extract was dried under a vacuum in a rotary evaporator (Eppendorf, Hamburg, Germany). The resultant ethanol extract of skullcap was further fractionated with different solvents, such as hexane, chloroform, ethyl acetate, butanol, and water. Each solvent collected was concentrated under vacuum in a rotary evaporator, and the concentrated fractions were lyophilized. The detailed method for obtaining the solvent fractions is shown in Figure 1.

Palmitic acid (PA), linoleic acid (LA), and linolenic acid (LNA) were purchased from Sigma-Aldrich (St. Louis, MO, USA), and PA, LA, LNA were used to investigate their effects on Th1/Th2 immune balance and allergen permeation across intestinal epithelium.

### 2.3. Sensitization with OVA and Preparation of Splenocyte Cultures

The mice were sensitized with 20 μg OVA (Grade VI; Sigma-Aldrich, St. Louis, MO, USA) adsorbed in 2 mg/mL Imject Alum (Pierce, Rockford, IL, USA) administered via an intraperitoneal injection on days 0 and 14. Splenocytes were prepared aseptically from each mouse. The homogenized single spleen cells were collected and treated with red blood cell (RBC)-lysing buffer (Sigma-Aldrich, St. Louis, MO, USA). The splenocytes were adjusted to 5 × 10^6^ cells/mL in RPMI 1640 medium and then cultured in 96-well tissue plate in the presence or absence of OVA (100 µg/mL/well) and the sample. The plates were incubated at 37 °C for 72 h in a humidified incubator with 5% (*v*/*v*) CO_2_. Cytokines in the supernatant were measured by Enzyme-linked immunosorbent assay (ELISA).

### 2.4. Measurement of Cytokine Levels Using ELISA

A cytokine assay kit was used to measure the cytokine levels (IFN-γ, IL-4, IL-5, IL-10 and IL-12 (BD PharMingen, San Diego, CA, USA) and IL-13 (R & D systems, Minneapolis, MN, USA)). All ELISAs were conducted in accordance with the manufacturer’s instructions. To measure IFN-γ, cell supernatant was diluted 6-fold, and other cytokines were detected using cell supernatant without dilution.

### 2.5. Measurement of Transepithelial Electrical Resistance

The human colon adenocarcinoma cell line, Caco-2 cells were obtained from American Type Culture Collection (ATCC, Rockvile, MD, USA). The Caco-2 cells were incubated at 37 °C in a humidified incubator with 5% (*v/v*) CO_2_. The cells were maintained in a 100-mm dish with Bulbecco’s modified Eagle’s medium (DMEM) containing 1000 mg/L glucose and supplemented with 10% fetal bovine serum (FBS), 1% non-essential amino acid (NEAA) 100 U/mL penicillin, and 100 µg/mL streptomycin. The cells were plated at a density of 2 × 10^5^ cells/mL on a 12-transwell or 24-well plate (Costar, Corning, New York, NY, USA) and allowed to grow for 3 weeks; the medium was changed every 2–3 days [4]. The cells were used between passages 25–45.

The monolayers of Caco-2 cells were used after incubating for three weeks. The integrity of the Caco-2 cell monolayers was checked by measuring the transepithelial electrical resistance (TEER) using a Millicell-ERS device (Millipore, Bedford, MA, USA). The TEER value primarily represents the TJ permeability of the intestinal epithelial monolayer [4,11]. When their TEER values were 300–500 Ω cm^−2^, the monolayers of Caco-2 cells were used in the experiment. The cell monolayers were washed twice with Hank’s balanced salt solution (HBSS) and pre-incubated for 30 min at 37 °C in a CO_2_ incubator to stabilize the cell monolayers. The Caco-2 monolayers were treated with each sample and bile salts (mixture of sodium cholate and sodium deoxycholate) for 60 min and then with OVA for 3 h at 37 °C. Following incubation, the TEER of the cell monolayers was measured, and the medium on the basolateral side of the monolayer, which contained the permeated OVA, was collected for ELISA.

Briefly, coating anti-OVA primary antibodies (ab17290) and horseradish peroxidase (HRP)-conjugated anti-OVA secondary antibodies (ab20415) for ELISA were purchased from Abcam (Cambridge, MA, USA). 96-well immunoplates were coated by overnight incubation at 4 °C with coating anti-OVA primary antibodies. After blocking for 2 h with 1% bovine serum albumin, the supernatant was incubated at room temperature for 2 h. After washing by Phosphate Buffered Saline (PBS) with 0.05% Tween 20, HRP-conjugated anti-OVA secondary antibodies were added for 1 h on plates, and then each well was reacted by 3,3’,5,5’-tetramethylbenzidine substrate solution in the dark. Finally, stop solution (2N sulfuric acid) was added, and then absorbance was measured at 450 nm using an Epoch microplate reader (BioTek, Winooski, VT, USA).

### 2.6. Analysis of Active Components Using High-Performance Liquid Chromatography (HPLC)

To investigate the compound in hexane fraction of skullcap extract, the hexane fraction was analyzed using the HPLC system JASCO 2000 PLUS (Jasco, Tokyo, Japan) equipped with a ZORBAX Rx-SIL-prep (9.4 × 250 mm; 5 µm; Agilent, Santa Clara, CA, USA) as normal phase column. The sample was prepared to 10 mg/mL in hexane solvent and then filtered 0.2 µm syringe filter (13 mm; Whatman, Maidstone, Kent, UK). The sample was eluted in a gradient with (A) hexane and (B) ethyl acetate at a flow rate of 5.0 mL/min for 45 min. The injection volume of sample was 10 µL and the sample was detected at 280 nm. Gradient elution was carried out using mobile phase hexane (A) and ethyl acetate (B) at 30 °C. The condition of gradient flow was set up to linear from 0 to 50% of B solution for 30 min and then progressed up to 100% of B solution for 5 min. The peaks were isolated with prep system using Fraction collector (FC 203B, Gilson, Middleton, WI, USA).

### 2.7. Fatty Acid Analysis

Fatty acids of skullcap hexane fraction were analyzed by the AOAC Official Methods 963.22 [13].

### 2.8. Statistical Analysis

Results are expressed as the mean ± standard deviation (SD). A statistical analysis was performed using the SAS statistical software package (SAS Institute, Cary, NC, USA). Differences between the experimental data were assessed by 1-way analysis of variance (ANOVA), followed by Duncan’s multiple-range test; a value of *p* < 0.05 was considered significant.

## 3. Results

### 3.1. Effects of Fractions Obtained from Skullcap Extracts

**Figure 2:**
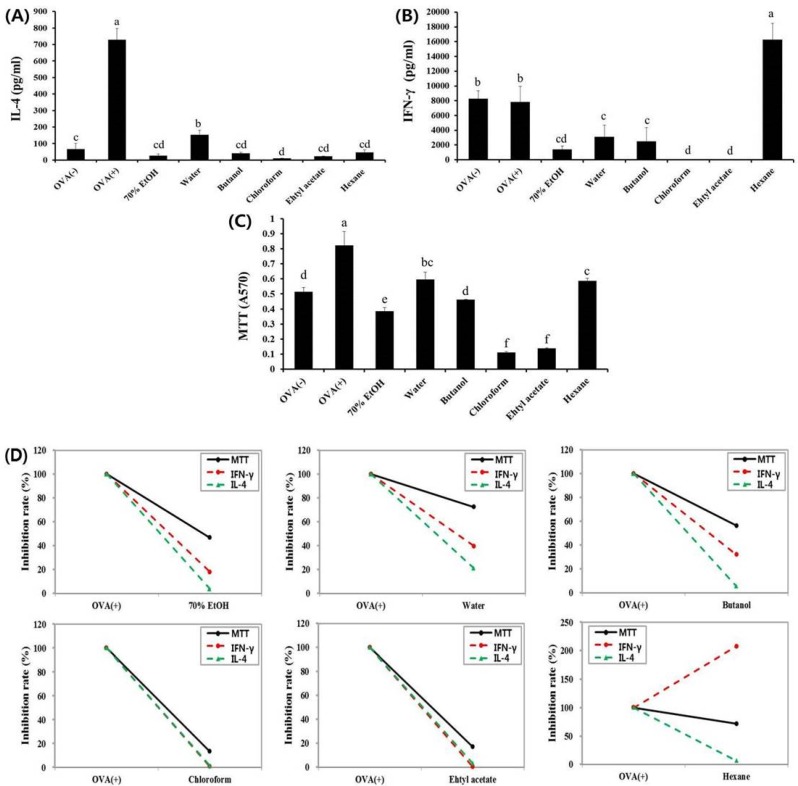
Effects of skullcap extract (ethanol, hexane, chloroform, ethyl acetate, butanol, and water) on ex vivo splenocytes from ovalbumin (OVA)-sensitized BALB/c mice. (**A**) Interleukin (IL)-4 and (**B**) Interferon (IFN)-γ were quantified by enzyme-linked immunosorbent assay (ELISA) using splenocytes; (**C**) Cell viability and cytotoxicity were measured by the 3-(4,5-dimethylthiazol-2-yl)-2,5-diphenyltetrazolium bromide (MTT) assay; (**D**) Inhibition rate (%). Values are presented as mean ± standard deviation (SD) (*n* = 4 in each group). Data were analyzed by analysis of variance (ANOVA), followed by Duncan’s multiple-range test. Data are statistically different (*p* < 0.05) among those columns with different symbols. The order of value is termed as order of alphabet.

In our previous study, we revealed that skullcap extract could ameliorate the symptoms of food allergy by regulating the immune balance between Th1 and Th2 responses [9]. To find the active fractions divided from the skullcap extract, we investigated the immunomodulatory effects of fractions on IFN-γ (Th1-mediated main cytokine) and IL-4 (Th2-mediated main cytokine) production in splenocytes isolated from OVA-sensitized mice. All the fractions significantly inhibited the OVA-induced increase in interleukin IL-4 production (Figure 2A). Furthermore, the production of IFN-γ was suppressed by four fractions: water, butanol, ethyl acetate, and chloroform (Figure 2B). The reduction of IL-4 and IFN-γ production by butanol, ethyl acetate, and chloroform fractions may have been due to the decrease in cell viability (Figure 2C). Interestingly, the hexane fraction strongly enhanced IFN-γ production and significantly suppressed IL-4 production by OVA. Unlike other fractions, the hexane fraction regulated abnormal immune responses without influencing cell viability or exhibiting cytotoxicity (Figure 2D). These results indicated that the effect of the hexane fraction was similar to that of the skullcap extract. Therefore, the active fraction in the skullcap extract, which regulates the Th1/Th2 immune balance, is most likely the hexane fraction.

### 3.2. Hexane Fraction of Skullcap Regulates Th1- and Th2-Mediated Cytokines in Splenocytes from OVA-Sensitized Mice

**Figure 3:**
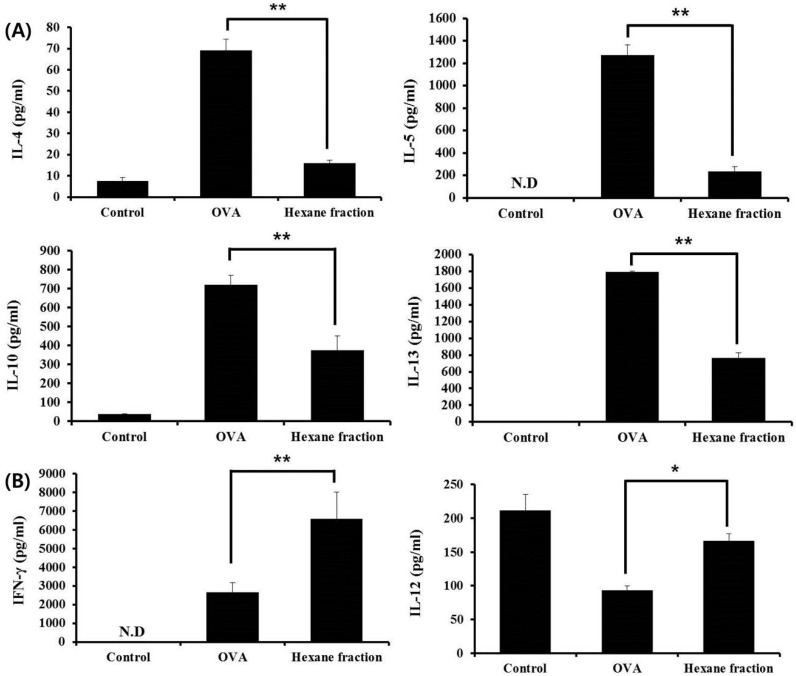
Effects of skullcap hexane fraction (50 µg/L on OVA-induced Th2 immune response ex vivo. (**A**) Th2-associated cytokines such as IL-4, IL-5, IL-10 and IL-13; (**B**) Th1-associated cytokines, such as IFN-γ and IL-12, were quantified by enzyme-linked immunosorbent assay (ELISA) using splenocytes. Values are presented as mean ± SD (*n* = 4 in each group). Data were analyzed by ANOVA, followed by Duncan’s multiple-range test. * *p* < 0.05, ** *p* < 0.01, significantly different from the OVA value.

We investigated whether the hexane fraction of skullcap regulates Th1- and Th2-associated cytokines in the splenocytes. Th2-associated cytokines, such as IL-4, IL-5, IL-10, and IL-13, and Th1-associated cytokines, such as IFN-γ and IL-12, were analyzed by ELISA. The hexane fraction significantly inhibited the secretion of IL-4, IL-5, IL-10, and IL-13, but enhanced the production of IFN-γ and IL-12 (Figure 3). These results indicated that the hexane fraction, isolated from the skullcap extract, could reverse abnormal allergic immune responses by reducing Th2 responses and increasing Th1 responses.

### 3.3. Fatty Acid Analysis of Skullcap Hexane Fraction

In general, the hexane fraction contains various non-polar components, such as lipids, fatty acids, and sterols. To reveal the active components in hexane fraction, we analyzed the profiles of fatty acids in the fraction using AOAC official method 963.22. Fatty acids detected in the hexane fraction were palmitic acid (C16:0), stearic acid (C18:0), oleic acid (C18:1), linoleic acid (LA (C18:2)), linolenic acid (C18:3), arachidic acid (C20:0), eicosadienoic acid (C20:2), behenic acid (C22:0), lignoceric acid (C24:0), and unknown compounds, and their proportions were 17.7%, 1.6%, 3.6%, 59.7%, 12.0%, 1.0%, 0.8%, 2.0%, 1.2% and 0.4%, respectively (Table 1). We focused on the main fatty acids in the hexane fraction, such as PA, LA, and LNA, using criteria based on contents of >10%.

### 3.4. LA, but Not PA or LNA, Regulates Th1/Th2 Immune Balance

**Figure 4:**
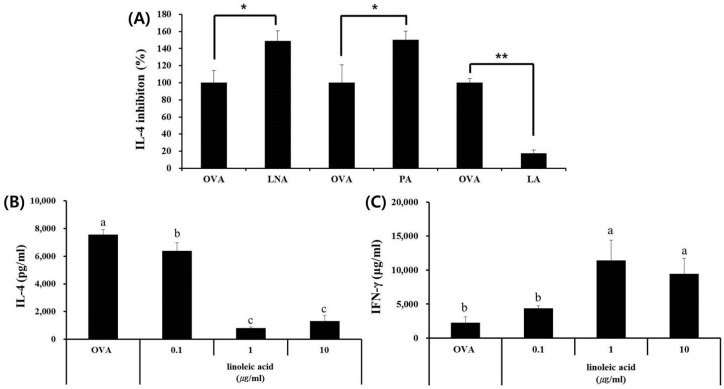
Effects of linoleic acid in skullcap hexane fraction on OVA-induced Th2 immune response ex vivo. (**A**) LNA, PA, and LA were evaluated the capacities for IL-4 inhibition rate (%); (**B**) IL-4 and (**C**) IFN-γ were quantified by ELISA using splenocytes. Values are presented as mean ± SD (*n* = 4 in each group). Data were analyzed by ANOVA, followed by Duncan’s multiple-range test. (**A**) * *p* < 0.05, ** *p* < 0.01, significantly different from the OVA value. (**B**,**C**) Data are statistically different (*p* < 0.05) among those columns with different symbols. The order of value is designated as alphabetical order.

We investigated the effects of three fatty acids (PA, LA, and LNA) selected from the analysis of the production of IL-4 and IFN-γ in splenocytes. Among the three fatty acids, LA significantly inhibited IL-4 production induced by OVA and enhanced IFN-γ production induced by OVA, but PA and LNA did not (Figure 4). Therefore, we confirmed that LA was a key component that could maintain the balance between Th1 and Th2 responses through immunomodulation.

### 3.5. Effects of Hexane Fraction and LA on Transepithelial Electrical Resistance in Caco-2 Cell Monolayers

**Figure 5:**
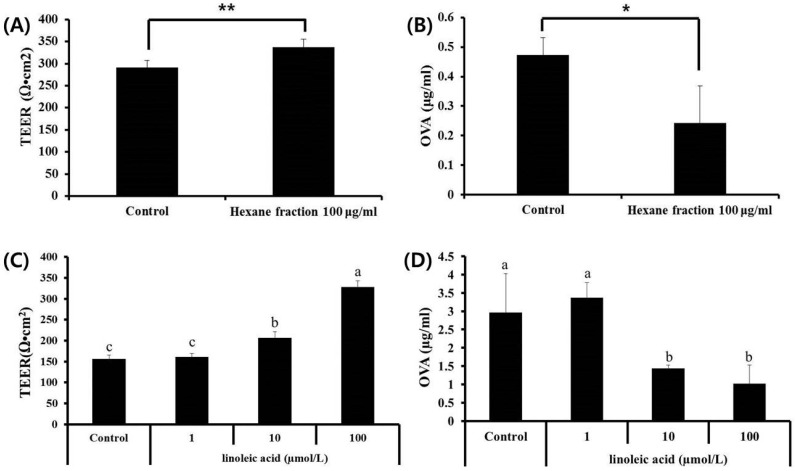
Effects of skullcap hexane fraction and key compound (linoleic acid) on TEER in Caco-2 cell monolayers and ovalbumin (OVA) flux in Caco-2 cell monolayers. (**A**,**B**) Effects of skullcap hexane fraction (100 µg/mL) on TEER value and OVA influx. (**C**,**D**) Effects of linoleic acid, a key compound in the hexane fraction, on TEER and OVA flux. Values are presented as mean ± SD (*n* = 4 in each group). Data were analyzed by ANOVA, followed by Duncan’s multiple-range test. (**A**,**B**) * *p* < 0.05, ** *p* < 0.01, significantly different from the control value. (**C**,**D**) Data are statistically different (*p* < 0.05) among those columns with different symbols. The order of value is designated as alphabetical order.

In our previous study, we also revealed that the hexane fraction of skullcap inhibited the permeation of OVA into the intestinal epithelial cells [4]. The results demonstrated that the hexane fraction could enhance intestinal barrier function. In this study, we investigated whether PA, LA, and LNA could enhance intestinal barrier function and inhibit the permeation of allergen by measuring TEER and OVA influx into the Caco-2 cell monolayers. After treatment with LA (1, 10 and 100 mmol/L), the TEER value considerably increased, and OVA influx decreased in a dose-dependent manner (Figure 5). However, treatment with PA and LNA did not change the TEER value and OVA influx (data not shown). These results indicated that LA was able to enhance intestinal barrier function by increasing TEER value and decreasing OVA influx into the intestinal epithelial cells, and is thus an active component.

## 4. Discussion

In the present study, we identified the active components and fractions in skullcap. We found that LA and the hexane fraction regulated allergic responses to maintain Th1/Th2 immune balance. Furthermore, the active components significantly diminished the permeation of allergen (OVA) by enhancing the intestinal barrier function.

The active ingredients of skullcap have been known to be polyphenols such as baicalin, baicalein, and wogonin. Baicalin induces Foxp3 expression in a dose-dependent manner (0–40 µmol/L), resulting in Treg differentiation [14], and reduces the permeability of the blood–brain barrier (BBB) by increasing the expression of claudin-5 and ZO-1 TJ proteins in the brain microvascular endothelial cells [15]. Furthermore, it was recently reported that baicalein effectively attenuated the symptoms of food allergy through its dual functions of the induction of CD4 + Foxp3 + T cells and enhancement of intestinal barrier function [7]. Wogonin also attenuated allergic immune responses by inhibiting Th2-associated responses [5]. To reveal active compounds of skullcap, we analyzed baicalein, baicalin, and wogonin in divided fractions. We confirmed that baicalein, baicalin, and wogonin were mainly detected in ethyl acetate and butanol fractions. Both fractions suppressed Th1/Th2-mediated immune responses. Therefore, we believe that the inhibitory activity of both fractions may have been derived from baicalein, baicalin, or wogonin.

To investigate the active compound in the hexane fraction, we analyzed the ingredients in the hexane fraction using HPLC, and then the 7 peaks were isolated with prep system. To reveal the functionality of the peaks, we examined whether each peak inhibits OVA-induced IL-4 production in splenocytes and increases TEER value in intestinal epithelial Caco-2 cell monolayers. However, IL-4 production and TEER value did not change (data not shown). Thus, we newly analyzed the profiles of fatty acids in the hexane fraction and found that LA could regulate IL-4, IFN-γ, and TEER value, and thus was an active compound in the hexane fraction of skullcap.

Intestinal epithelium plays a major role and has various functions such as nutrition absorption, provision of a physical barrier, and interaction with immune cells [16,17]. Among these functions, the reinforcement of the intestinal barrier is important in preventing inflammatory and infectious disorders caused by penetration of antigens, allergens (immunogens), and toxic substances in the immune system. TJ, which is the most intercellular structure in epithelial cells, can maintain the barrier function of intact intestinal epithelium [18,19]. It is composed of various proteins, such as occludins, zonula occludens (zo-1), and claudins. Maintaining the barrier function and TJ may play a major role in the treatment of chronic inflammatory bowel disease (IBD) [16,20]. Furthermore, up-regulatory effects of food and natural materials on the expression and assembly of TJ proteins could inhibit allergen permeation and enhance the barrier function of the epithelium. For example, trachelogenin from *Trachelospermi caulis* extract has been reported to attenuate the symptoms of OVA-induced food allergy by enhancing the expression of occludin [21]. In other words, the regulation of TJ by food and natural materials might be able to contribute to ameliorating allergic disorders, because TJ could block the permeations of allergens from the external environment into the tissues. In the present study, we found that the hexane fraction and its active component LA enhanced the intestinal barrier and reduced the permeation of allergen OVA. We suggest that the hexane fraction of skullcap extract and LA are able to contribute to attenuating allergic diseases such as allergic rhinitis, asthma, and food allergy. In future studies, we will investigate those TJ proteins that are up-regulated by the hexane fraction and LA, and their enhancing effects when applied in an animal model of allergic disorder.

However, the enhancement of intestinal barrier function alone cannot completely block antigens (immunogens) from entering the tissues, and the penetrating antigens may induce abnormal immune responses. To prevent these immune disorders completely, dual functions, such as enhancement of physical barrier and immunomodulatory effects, may be essential. In a previous study, we revealed the dual functions of baicalein (enhancement of intestinal barrier and induction of Treg cells) [7], and in this study, our results demonstrated that LA from the hexane fraction of skullcap regulated steady immune responses and enhanced the intestinal barrier function.

Regulating T cell-mediated immune responses were effective for improving allergic responses. To evaluate anti-allergic effects on T cell-mediated immune responses, we used an ex vivo experiment system using splenocytes. Many studies have revealed anti-allergic effects and mechanisms of hydrolysates, probiotics, and natural products using this system [22,23,24]. For example, *Lactobacillus pentosus* KF340 ameliorated the symptoms of atopic dermatitis induced by house dust mite [25]. The *L. pentosus* KF340 increased IL-10 secretion and decreased IL-4 production through induction of IL-10-producing B cells. Our results showed that the hexane fraction of skullcap extract and LA regulated Th1/Th2 immune balance by increasing IFN-γ and reducing IL-4. However, in terms of Th1/Th2 immune balance, it is not yet known whether IL-4 was reduced first in the samples, or IL-4 was decreased by the increase of IFN-γ. This mechanism will be explored in further study.

LA (18:2*n*-6) is subsequently converted to γ-LA (GLA, 18:3*n*-6) and dihomo-γ-LA (DGLA, 20:3*n*-6), and further into arachidonic acid (AA, 20:4*n*-6). DGLA is a precursor of eicosanoids, such as the prostaglandin (PG) 1-series, and has anti-inflammatory, anti-allergic, and anti-thrombotic activities. Studies have suggested that dietary DGLA, GLA was effective against inflammation and platelet aggregation [26,27,28]. In particular, DGLA, GLA, which has anti-allergic effects, such as for atopic dermatitis in NC/Nga mice and humans, suppressed clinical severity of skin lesions [29,30,31]. Thus, we believe LA is a precursor to DGLA, GLA, and the hexane fraction of skullcap is a useful anti-inflammatory agent, and may be effective against immune diseases caused by immune system imbalance.

## 5. Conclusions

In conclusion, we demonstrated that LA from the hexane fraction of skullcap extract inhibited the permeation of food allergen across the intestinal epithelium and regulated the allergic immune response by maintaining the balance between Th1 and Th2 cytokines. Therefore, we suggest that LA could be used in preventive and/or therapeutic approaches to allergic disorders.

## Figures and Tables

**Table 1:** Principal components of fatty acid in the skullcap hexane fraction.

Chemical Formula	Fatty Acid	Composition Ratio (%) per 100 g Fat
C16:0	Palmitic acid	17.7
C18:0	Stearic acid	1.6
C18:1	Oleic acid	3.6
C18:2	Linoleic acid	59.7
C18:3	Linolenic acid	12.0
C20:0	Arachidic acid	1.0
C20:2	Eicosadienoic acid	0.8
C22:0	Behenic acid	2.0
C24:0	Lignoceric acid	1.2
	Unknown	0.4
Total		100.0

## References

[1] Li-Weber M. (2009). New therapeutic aspects of flavones: The anticancer properties of cutellaria and its main active constituents Wogonin, Baicalein and Baicalin. Cancer Treat. Rev..

[2] Zhang Z., Lian X.Y., Li S., Stringer J.L. (2009). Characterization of chemical ingredients and anticonvulsant activity of American skullcap (*Scutellaria lateriflora*). Phytomedicine.

[3] Gao J., Sanchez-Medina A., Pendry B.A., Hughes M.J., Webb G.P., Corcoran O. (2008). Validation of a HPLC method for flavonoid biomarkers in sckullcap (Scutellaria) and its use to illustrate wide variability in the quality of commercial tinctures. J. Pharm. Sci..

[4] Shin H.S., Bae M.J., Jung S.Y., Shon D.H. (2013). Inhibitory effect of skullcap (*Scutellaria baicalensis*) extract on ovalbumin permeation in vitro and in vivo. Food Chem..

[5] Shin H.S., Bae M.J., Choi D.W., Shon D.H. (2014). Skullcap (*Scutellaria baicalensis*) extract and its active compound, wogonin, inhibit ovalbumin-induced Th2-mediated response. Molecules.

[6] Zou Y., Dai S.X., Chi H.G., Li T., He Z.W., Wang J., Ye C.G., Huang G.L., Zhao B., Li W.Y. (2015). Baicalin attenuates TNBS-induced colitis in rats by modulating the Th17/Treg paradigm. Arch. Pharm. Res..

[7] Bae M.J., Shin H.S., See H.J., Jung S.Y., Kwon D.A., Shon D.H. (2016). Baicalein induces CD4 + Foxp3 + T cells and enhances intestinal barrier function in a mouse model of food allergy. Sci. Rep..

[8] Romagnani S. (2004). Immunologic influences on allergy and the Th1/Th2 balance. J. Allergy Clin. Immunol..

[9] Shin H.S., Bae M.J., Jung S.Y., Shon D.H. (2014). Preventive effects of skullcap (*Scutellaria baicalensis*) extract in a mouse model of food allergy. J. Ethnopharmacol..

[10] Kaminogawa S., Hachimura S., Nakajjima-adachi H., Totsuka M. (1999). Food allergens and mucosal immune systems with special reference to recognition of food allergens by gut-associated lymphoid tissue. Allergol. Int..

[11] Kobayashi S., Watanabe J., Fukushi E., Kawabata J., Nakajima M., Watanabe M. (2003). Polyphenols from some foodstuffs as inhibitors of ovalbumin permeation through Caco-2 cell monolayers. Biosci. Biotechnol. Biochem..

[12] Mine Y., Zhang J.W. (2003). Surfactants enhance the tight-junction permeability of food allergens in human intestinal epithelial Caco-2 cells. Int. Arch. Allergy Immunol..

[13] AOAC International (2000). Methyl esters of fatty acids in oils and fats. AOAC official method 963.22. Official Methods of Analysis.

[14] Yang J., Yang X., Li M. (2012). Baicalin, a natural compound, promotes regulatory T cell differentiation. BMC Complement. Altern. Med..

[15] Zhu H., Wang Z., Xing Y., Gao Y., Ma T., Lou L., Lou J., Gao Y., Wang S., Wang Y. (2012). Baicalin reduces the permeability of blood-brain barrier during hypoxia in vitro by increasing the expression of tight junction proteins in brain microvascular endothelial cells. J. Ethnopharmacol..

[16] Koch S., Nusrat A. (2009). Dynamic regulation of epithelial cell fate and barrier function by intercellular junctions. Ann. N. Y. Acad. Sci..

[17] Macdonald T., Monteleone G. (2005). Immunity, inflammation, and allergy in the gut. Science.

[18] Neurath M.F., Finotto S., Glimcher L.H. (2002). The role of Th1/Th2 polarization in mucosal immunity. Nat. Med..

[19] Gassler N., Rohr C., Schneider A., Kartenbeck J., Bach A., Obermuller N., Fotto H., Autschbach F. (2001). Inflammatory bowel disease is associated with changes of enterocytic junctions. Am. J. Physiol. Cell Physiol..

[20] Li N., Gu L., Qu L., Gong J., Li Q., Zhu W., Li J. (2010). Berberine attenuates pro-inflammatory cytokine-induced tight junction disruption in an in vitro model of intestinal epithelial cells. Eur. J. Pharm. Sci..

[21] Shin H.S., Bae M.J., Jung S.Y., See H.J., Kim Y.T., Do J.R., Back S.Y., Choi S.W., Shon D.H. (2015). Enhancing effect of trachelogenin from *Trachelospermi caulis* extract on intestinal barrier function. Biol. Pharm. Bull..

[22] Lozano-Ojalvo D., Molina E., López-Fandiño R. (2016). Hypoallergenic hydrolysates of egg white proteins modulate allergen responses induced ex vivo on spleen cells from sensitized mice. Food Res. Intern..

[23] Holvoet S., Zuercher A.W., Julien-Javaux F., Perrot M., Mercenier A. (2013). Characterization of candidate anti-allergic probiotic strains in a model of Th2-skewed human peripheral blood mononuclear cells. Int. Arch. Allergy Immunol..

[24] Park E.J., Kim B., Eo H., Park K., Kim Y., Lee H.J., Son M., Chang Y.S., Cho S.H., Kim S. (2005). Control of IgE and selective T(H)1 and T(H)2 cytokines by PG102 isolated from *Actinidia arguta*. J. Allergy Clin. Immunol..

[25] Umeda-Sawada R., Fujiwara Y., Ushiyama I., Sagawa S., Morimitsu Y., Kawashima H., Ono Y., Kiso Y., Matsumoto A., Seyama Y. (2006). Distribution and metabolism of dihomo-γ-linolenic acid (DGLA, 20:3*n*-6) by oral supplementation in rats. Biosci. Biotechnol. Biochem..

[26] Bae M.J., Kim H.K., Lim S., Lee S.Y., Shin H.S., Kim J.E., Im S.H., Kim S.Y. (2016). *Lactobacillus pentosus* KF340 alleviates house dust mite-induced murine atopic dermatitis via the secretion of IL-10-producing splenic B10 cells. J. Funct. Foods.

[27] Smith D.L., Willis A.L., Nguyen N., Conner D., Zahedi S., Fulks J. (1989). Eskimo plasma constituents, dihomo-γ-linolenic acid, eicosapentaenoic acid and docosahexaenoic acid inhibit the release of atherogenic mitogens. Lipids.

[28] Nakamura N., Hamazaki T., Taki H., Yamazaki K., Kobayashi M. (1993). Intravenous infusion of tridihomo-γ-linoleoyl-glycerol reduces leukotriene B4 production in the rat and rabbit. Clin. Sci..

[29] Kawashima H., Tateishi N., Shiraishi A., Teraoka N., Tanaka T., Tanaka A., Matsuda H., Kiso Y. (2008). Oral administration of dihomo-γ-linolenic acid prevents development of atopic dermatitis in NC/Nga Mice. Lipids.

[30] Van Gool C.J., Thijs C., Henquet C.J., van Houwelingen A.C., Dagnelie P.C., Schrander J., Menheere P.P., van den Brandt P.A. (2003). γ-Linolenic acid supplementation for prophylaxis of atopic dermatitis-a randomized controlled trial in infants at high familial risk. Am. J. Clin. Nutr..

[31] Kawamura A., Ooyama K., Kojima K., Kachi H., Abe T., Amano K., Aoyama T. (2011). Dietary supplementation of Gamma-Linolenic acid improves skin parameters in subjects with dry skin and mild atopic dermatitis. J. Oleo Sci..

